# Predicting outcomes in patients with aortic stenosis using machine learning: the Aortic Stenosis Risk (ASteRisk) score

**DOI:** 10.1136/openhrt-2022-001990

**Published:** 2022-05-31

**Authors:** Mayooran Namasivayam, Paul D Myers, John V Guttag, Romain Capoulade, Philippe Pibarot, Michael H Picard, Judy Hung, Collin M Stultz

**Affiliations:** 1 Division of Cardiology, Massachusetts General Hospital, Harvard Medical School, Boston, Massachusetts, USA; 2 Department of Electrical Engineering and Computer Science, Massachusetts Institute of Technology, Cambridge, Massachusetts, USA; 3 Computer Science and Artificial Intelligence Laboratory, Massachusetts Institute of Technology, Cambridge, Massachusetts, USA; 4 l’institut du thorax, CHU Nantes, CNRS, INSERM, University of Nantes, Nantes, France; 5 Cardiology, Quebec Heart and Lung Institute, Laval University, Quebec City, Quebec, Canada; 6 Research Laboratory of Electronics, Massachusetts Institute of Technology, Cambridge, Massachusetts, USA

**Keywords:** Artificial intelligence, machine learning, aortic stenosis, clinical outcome, echocardiography

## Abstract

**Objective:**

To use echocardiographic and clinical features to develop an explainable clinical risk prediction model in patients with aortic stenosis (AS), including those with low-gradient AS (LGAS), using machine learning (ML).

**Methods:**

In 1130 patients with moderate or severe AS, we used bootstrap lasso regression (BLR), an ML method, to identify echocardiographic and clinical features important for predicting the combined outcome of all-cause mortality or aortic valve replacement (AVR) within 5 years after the initial echocardiogram. A separate hold out set, from a different centre (n=540), was used to test the generality of the model. We also evaluated model performance with respect to each outcome separately and in different subgroups, including patients with LGAS.

**Results:**

Out of 69 available variables, 26 features were identified as predictive by BLR and expert knowledge was used to further reduce this set to 9 easily available and input features without loss of efficacy. A ridge logistic regression model constructed using these features had an area under the receiver operating characteristic curve (AUC) of 0.74 for the combined outcome of mortality/AVR. The model reliably identified patients at high risk of death in years 2–5 (HRs ≥2.0, upper vs other quartiles, for years 2–5, p<0.05, p=not significant in year 1) and was also predictive in the cohort with LGAS (n=383, HRs≥3.3, p<0.05). The model performed similarly well in the independent hold out set (AUC 0.78, HR ≥2.5 in years 1–5, p<0.05).

**Conclusion:**

In two separate longitudinal databases, ML identified prognostic features and produced an algorithm that predicts outcome for up to 5 years of follow-up in patients with AS, including patients with LGAS. Our algorithm, the Aortic Stenosis Risk (ASteRisk) score, is available online for public use.

**WHAT IS ALREADY KNOWN ON THIS TOPIC**

- Current guideline criteria used to make clinical decisions in aortic stenosis (AS) are limited in number and are particularly challenging to apply in particular patient subgroups, namely low-gradient AS (LGAS). Machine learning (ML) models in medicine are traditionally challenging to apply at the bedside.

**WHAT THIS STUDY ADDS**

- We show that application of an ML algorithm to combined echocardiographic and clinical data in patients with AS can provide good predictive capability for mortality and aortic valve replacement up to 5 years post echocardiography, and the algorithm performs well inthe clinically challenging patient subgroup LGAS. Furthermore, the algorithm developed is explainable and interpretable at a clinical level and uses few inputs that can be easily incorporated at the bedside.

**HOW THIS STUDY MIGHT AFFECT RESEARCH, PRACTICE AND/OR POLICY**

- ML algorithms applied to echocardiographic and clinical data can yield valuable risk-predictive capability using few inputs at the bedside, extending the use of ML in AS beyond phenotyping and diagnosis and making use of available data that current guidelines for AS ignore.

## Introduction

Aortic stenosis (AS) is increasingly prevalent and is associated with increased mortality, even when moderate in severity.^1 2^ Identifying patients with AS who are at increased risk of death is challenging because of the complex interplay of multiple factors that determine risk. Conventional risk assessments use few echocardiographic criteria, namely aortic valve area, mean transvalvular gradient and peak transvalvular velocity.^3^ This is clearly incomplete^4^—two patients with identical valve gradients can have very different risks based on other interacting, and unaccounted for, features.

Machine learning approaches can provide enhanced analysis of otherwise wasted data that are acquired as part of routine clinical care and can be used to estimate clinical risks. Such approaches applied to electronic health record (EHR) data have been used to accurately predict clinical outcomes.^5–7^ Yet, findings are not always explainable and are not currently practically usable at the bedside. No study to date has evaluated clinical risk prediction in AS using machine learning analysis of combined echocardiographic and clinical data.

We sought to develop an explainable model^8^ that could be readily used at the bedside through a simple risk calculator requiring a few easily available input features. We also aimed to have a model that would be effective in the subgroup of patients with low-gradient aortic stenosis (LGAS) in whom clinical decision-making is extremely challenging.^9 10^

## Methods

### Primary cohort

This study analysed a previously described longitudinal ‘loyalty’ cohort of patients (n=1130) with moderate or greater severity AS defined by echocardiographic aortic valve area ≤1.5 cm^2^ and mean gradient ≥20 mm Hg.^11^ All patients had longitudinal primary care at the Massachusetts General Hospital (MGH), Boston, Massachusetts, USA, with complete outcome follow-up until either death or the end of the study period (study period commencing 2006 and ending 31 December 2017) with no loss to follow-up. Patients with aortic dissection, coarctation, high left ventricular outflow tract velocity (≥1.6 m/s, which might indicate subaortic restriction to flow) or moderate or greater aortic or mitral regurgitation were excluded. One patient from the originally described cohort^11^ was excluded from this study because the exact date of death was not available.

### Data preprocessing

Patients in the primary cohort were described by a total of 316 (62 clinical and 254 echocardiographic) features. We excluded features that had missing data in greater than 50% of the patients leaving 69 features (online supplemental table 1). Of these remaining 69 features, the missing data rate was very low overall with most variables complete (median missing data per variable 0.15%, mode 0% and mean (skewed data) 5.6%). Categorical variables with more than two categories were binarised (ie, recoded to have only two categories). For example, aortic valve morphology was originally coded as either tricuspid, bicuspid, vertical bicuspid or horizontal bicuspid. We grouped all bicuspid categories into a single category, leaving only two categories—bicuspid or tricuspid. We also binarised the aortic valve area (≤1.0 cm^2^ coded as 1), the mean pressure gradient (≥40 mm Hg coded as 1) and the flow rate (≤210 mL/s coded as 1). All continuous features were min–max normalised to fall between 0 and 1, inclusive. This was done to facilitate all input variables being on the same scale (ie, between 0 and 1) helping to interpret the coefficients arising from a logistic regression model.

10.1136/openhrt-2022-001990.supp1 Supplementary data

Missing data were handled in one of two ways. For some binary variables, we assumed that a missing entry for each of these fields indicated that the result was normal, and considered not worth reporting, by the reporting clinician. We refer to these features as *presumed normal* and replaced these missing entries with the appropriate code for normal for that variable. For aortic valve morphology, for example, a blank was assumed to be normal tricuspid morphology. For features where a missing entry could not be presumed normal, we imputed values using a multivariate imputation method.^12^ Thirty-one features had missing data requiring imputation.

### Feature selection

Bootstrap lasso (Least Absolute Shrinkage and Selection Operator) regression (BLR) was used for feature selection.^13 14^ In this method, an L1-regularised logistic regression model is trained using repeated rounds of bootstrapping. Lasso regression models have the property that many of the feature weights in the model are forced to zero, leaving only the most important features in the final model. Since the features that are selected by lasso regression may differ depending on the precise dataset used for training, we only use features that are consistently retained (ie, have non-zero weights) after many bootstrap iterations. We used 100 bootstrap splits, stratified by outcome, in which 80% of the data were used for training. Each bootstrap split consisted of a different set of patients randomly sampled with replacement from the entire dataset. Features with non-zero weight in at least 85% of the bootstrapped splits were retained for further model development. The regularisation parameter for the L1 regression was chosen using threefold cross validation, with the parameter being chosen separately for each bootstrap split (see online supplemental information).

The choice of BLR threshold (85% in this case) entails a trade-off between which features are deemed important according to domain knowledge and the number of features selected. Based on prior work,^12^ we initially used a value of 90%, but found that the mean gradient, a feature that is known to have prognostic significance, was not selected. We elected to lower the threshold to 85% to include this classically prognostic feature (see Discussion section).

### Aortic Stenosis Risk (ASteRisk) score

We trained a logistic regression model to predict a combined outcome consisting of all-cause mortality or aortic valve replacement. As we hypothesised that patients who received an aortic valve replacement (AVR) were deemed to be at high risk of death if they did not receive a valve replacement, we included AVR in the combined outcome to improve our ability to identify patients who are at high risk of death; that is, AVR was treated as an aborted death event.

**Figure 1:**
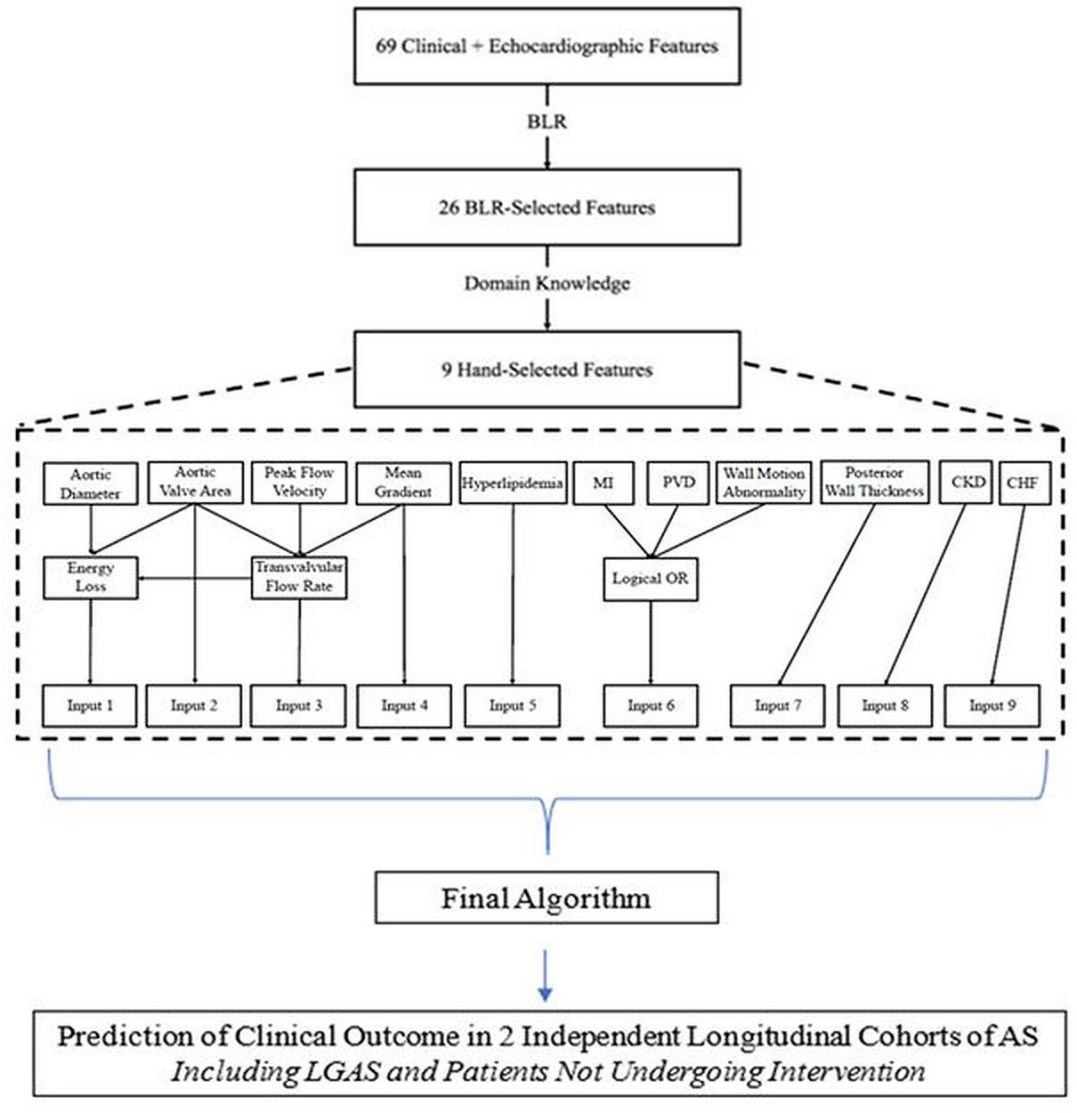
Flowchart describing feature selection and model construction. AS, aortic stenosis; CHF, congestive heart failure; CKD, chronic kidney disease; LGAS, low-gradient aortic stenosis; MI, myocardial infarction; PVD, peripheral vascular disease.

From 69 input features, we obtained 26 features from BLR. We then selected a subset of these 26 features to form a parsimonious set that could be readily entered into an online risk calculator. In reducing the feature set size, we kept features that were readily available to clinicians, are measured by current practice guidelines and appeared in the most bootstrap splits (figure 1). The resulting 9 features were used to train an L2-regularised logistic regression model to predict the combined outcome of death/AVR. Model performance, by area under the receiver operating characteristic curve (AUC) analysis, was compared among models containing 69, 26 and 9 features. For transvalvular flow rate, we used an empiric cut-off of 230 mL/s in the early BLR but chose to refine the threshold to 210 mL/s in the final model construction, to be consistent with the recent identification of this threshold flow rate at which aortic valve area becomes prognostic.^11^ The thresholds for the aortic valve area and mean gradient were the same as those used in the BLR analysis.

Bootstrapping was done to obtain statistical measures of performance. A total of 10 stratified bootstrap splits (80% training, 20% testing) were performed for evaluation, and the results are reported over the bootstrapped test sets. We used AUC analysis as well as the 1–5 year HRs. For the HRs, we chose the upper quartile of risk to denote the high-risk subgroup. Cox proportional hazards models were used for time-to-event analyses. CIs for the AUCs were calculated by mean±2 SEs of the AUCs across the 10 bootstrap splits. All CIs in the primary cohort are reported on these 10 bootstrap splits. AUCs were compared using paired t-test.

### Validation cohort

The validation cohort consisted of 540 patients with AS from Laval University, Quebec, Canada, who also had longitudinal follow-up and similar inclusion and exclusion criteria. This cohort came from a previously reported subset of patients,^11^ which itself was drawn from a prior study cohort that recruited 1999–2007.^15^ There were differences in coding of two features between the primary cohort (MGH) and validation cohort (Laval). First, the MGH dataset encodes a history of congestive heart failure without specifying the New York Heart Association (NYHA) class, while the validation cohort lists the NYHA class for each patient. To unify the coding structure, in the validation cohort we coded NYHA class ≤2 as zero and NYHA class >2 as one. Second, peripheral vascular disease at baseline and regional wall motion abnormalities were not available in the Laval dataset. Since the MGH-based model uses the ‘logical or’ of ‘myocardial infarction, peripheral vascular disease, or abnormal wall motion’ to represent significant atherosclerotic burden, we used only myocardial infarction in the Laval dataset for this feature.

To impute missing data, a second multivariate imputation model was trained on the final set of 9 features from the primary cohort and applied to the validation cohort.

CIs for the AUCs were calculated by randomly sampling 20% of the dataset, computing the AUC in that sample, and calculating the SD over 10 such random samples. The CIs were computed using the mean±2 SEs as in the primary cohort.

### Baseline risk model

For comparison, we also constructed a baseline risk model using conventional clinical features: (1) mean transvalvular gradient, (2) aortic valve area, (3) age and (4) left ventricular ejection fraction.^6 16^ These four features were used as input to an L2-regularised logistic regression model that was trained and tested in the same way as the ASteRisk score was, as described above.

Statistical analyses were performed using Python 3.6.8 (Python Software Foundation, Wilmington, Delaware, USA), MATLAB 2017a (MathWorks, Natick, Massachusetts, USA) and SPSS V.26 (IBM, Armonk, New York, USA).

### Ethics approval

Studies complied with the Declaration of Helsinki and were approved by the respective Institutional Review Board and Ethics Committees for the primary and validation cohorts (human ethics approval given by MGH Institutional Review Board Approval ID 2009P000122 and Quebec Heart and Lung Institute). Informed consent was not required.

## Results

Descriptive statistics for the primary and validation cohorts are shown in table 1. Of the 69 features available for each patient, 26 were selected by the algorithm. This was further reduced to 9 features (box 1).

**Table 1:** Descriptive statistics

	Primary cohort (n=1130) Mean±SD or n (%)	Validation cohort (n=540) Mean±SD or n (%)	Significance*
Age	76.7±11.0	69.5±13.2	<0.001
Female sex	453/1130 (40.1)	230/540 (43.6)	NS (0.17)
White race	1041/1130 (92.1)	–	–
Aortic valve area (cm^2^)	1.04±0.27	0.96±0.27	<0.001
Mean gradient (mm Hg)	29.5±13.7	29.5±15.9	NS (0.17)
Peak gradient (mm Hg)	51.8±22.0	49.9±24.9	<0.001
Transvalvular flow rate (mL/s)	243.5±52.1	226.4±54.6	<0.001
Energy loss	19.4±10.6	20.4±12.6	<0.001
Posterior wall thickness (mm)	11.3±1.9	10.8±2.0	<0.001
Left ventricular ejection fraction (%)	65.3±12.1	62.3±12.4	0.02
Myocardial infarction	235/1130 (20.8)	164/540 (30.3)	<0.001
Peripheral vascular disease	447/1130 (39.6)	–	–
Regional wall motion abnormality	133/1130 (11.8)	–	–
Hyperlipidaemia	1011/1130 (89.5)	320/540 (59.3)	<0.001
Chronic kidney disease	296/1130 (26.2)	96/540 (17.8)	<0.001
Heart failure	358/1130 (31.7)	121/540 (22.4)	0.002
Mortality rate	395/1130 (35.0)	252/540 (51.3)	<0.001
AVR rate	354/1130 (31.3)	344/540 (63.7)	<0.001
LGAS mortality rate	162/383 (42.3)	196/316 (62.0)	<0.001
LGAS AVR rate	126/383 (32.9)	189/316 (59.8)	<0.001
Mortality rate in the absence of intervention (AVR)	340/776 (43.8)	138/196 (70.4)	<0.001

*Using Mann-Whitney U test and χ^2^ test, two-sided significance.

AVR, aortic valve replacement; LGAS, low-gradient aortic stenosis; NS, not significant.

Box 1 **Features selected in final algorithm**

**Model features**

Features identified by bootstrap lasso regression

1. Transvalvular flow rate*
2. Mean gradient
3. Aortic valve area
4. Race
5. Heart failure
6. Peripheral vascular disease
7. Hyperlipidaemia
8. Myocardial infarction
9. Beta-blocker
10. Angiotensin receptor blocker
11. Nitrate
12. Statin
13. Antiplatelet
14. Oral anticoagulant
15. Chronic kidney disease (CKD)
16. Posterior wall thickness
17. Regional wall motion abnormality
18. CKD stage change event
19. Energy loss†
20. Gender
21. Hypertension
22. Coronary artery disease
23. Potassium sparing diuretics
24. Aortic insufficiency
25. Mitral insufficiency
26. Energy loss coefficient‡

**Final 9 features**

1. Aortic valve area
2. Mean gradient
3. Transvalvular flow rate†
4. Energy loss†
5. Posterior wall thickness
6. Heart failure
7. Myocardial infarction OR peripheral vascular disease OR regional wall motion abnormality
8. Hyperlipidaemia
9. CKD

### Primary cohort

The minimum sample size required for a model with 9 features, an event fraction of 0.35, and a maximum root mean squared prediction error of 0.05 is 450, which is met in this analysis.^17^

The ASteRisk score, which was trained on all 9 features, had an AUC of 0.74 (95% CI 0.73 to 0.76) for the combined outcome. The discriminatory ability of this model was superior to that of the baseline model (table 2). The ASteRisk score reliably identified patients at high risk of death at years 2–5 of follow-up, while the baseline model identified high-risk patients only at year 1 (table 3). Moreover, the discriminatory ability of the ASteRisk score was similar to that of a model trained with all 69 features (AUC of full model 0.75 with 95% CI 0.73 to 0.77, p=0.37 vs ASteRisk score). There was also no significant difference to the performance of the ASteRisk score to the intermediary model with 26 features.

**Table 2:** Discriminatory ability of baseline and ASteRisk score for the combined outcome of death/AVR in both the primary and validation cohorts

Combined outcome death/AVR*		
Model	AUC	P value
**Primary cohort**		
Baseline	0.69 (0.68,0.71)	0.000042
ASteRisk score	0.74 (0.73, 0.76)	
**Validation cohort**		
Baseline	0.75 (0.73, 0.77)	0.02
ASteRisk score	0.78 (0.77,0.80)	

*For death alone, the AUC results are: primary cohort: ASteRisk score 0.66±0.04, baseline: 0.61±0.04; validation cohort: ASteRisk score 0.61±0.05, baseline: 0.59±0.03.

ASteRisk score, Aortic Stenosis Risk score; AUC, area under the receiver operating characteristic curve; AVR, aortic valve replacement.

**Table 3:** HRs for death in the primary cohort, LGAS subset and subset of patients in whom no intervention was performed

HRs for death in the primary cohort (n=1130)					
Model	1-Year HR (95% CI)	2-Year HR (95% CI)	3-Year HR (95% CI)	4-Year HR (95% CI)	5-Year HR (95% CI)
Baseline	2.8 (1.2 to 6.9)	1.7 (0.9 to 3.2)	1.5 (0.8 to 2.6)	1.6 (0.9 to 2.6)	1.6 (1.0 to 2.5)
ASteRisk score	2.2 (0.9 to 5.5)	2.1 (1.1 to 4.0)	2.1 (1.2 to 3.7)	2.0 (1.2 to 3.3)	2.0 (1.3 to 3.2)
**Patients with LGAS (n=383)**					
Baseline	10.9 (2.3 to 62.4)	3.7 (1.4 to 9.7)	3.5 (1.5 to 8.3)	3.7 (1.7 to 8.2)	3.6 (1.7 to 7.7)
ASteRisk score	3.0 (0.7 to 13.4)	3.5 (1.3 to 9.5)	4.1 (1.7 to 10.0)	3.5 (1.6 to 7.6)	3.3 (1.6 to 7.0)
**Patients without intervention (n=776)**					
Baseline	4.8 (1.9 to 12.0)	2.9 (1.4 to 5.7)	2.5 (1.3 to 4.6)	2.5 (1.4 to 4.4)	2.4 (1.4 to 4.3)
ASteRisk score	3.0 (1.2 to 7.7)	2.9 (1.5 to 5.7)	3.0 (1.7 to 5.5)	2.8 (1.6 to 4.8)	2.8 (1.7 to 4.9)

P value <0.05, where HR (95% CI) does not cross/include 1. Results are from 10 bootstrapped test sets. HRs are calculated using the upper quartile of risk.

ASteRisk score, Aortic Stenosis Risk score; LGAS, low-gradient aortic stenosis.

For patients with LGAS (n=383), both the baseline and ASteRisk score reliably identified those at high risk of death for years 2–5, with the baseline model also being predictive at year 1 (table 3). Among the cohort of patients who did not receive an intervention (n=776), both models had statistically significant HRs for years 1–5 (table 3, see also online supplemental table 2).

**Figure 2:**
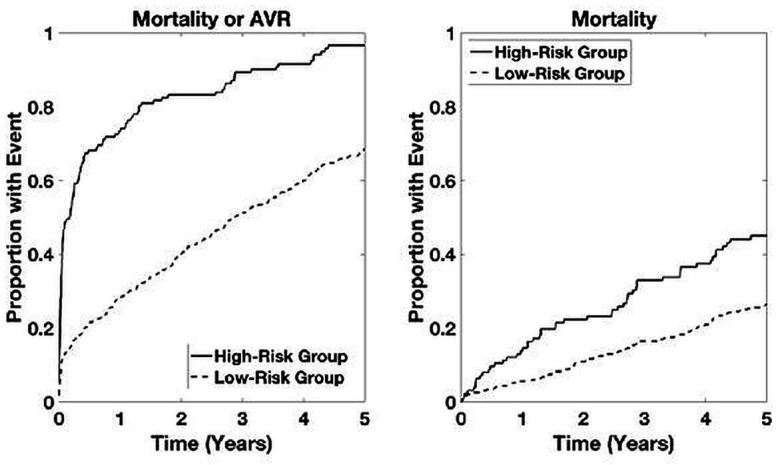
Time-to-event analysis based on Aortic Stenosis Risk score (ASteRisk score) in patients with aortic stenosis within the primary cohort. High-risk group determined by upper quartile of ranked risk. Curves are averaged over 10 bootstrapped test sets. p<0.05 high-risk group versus others.

**Figure 3:**
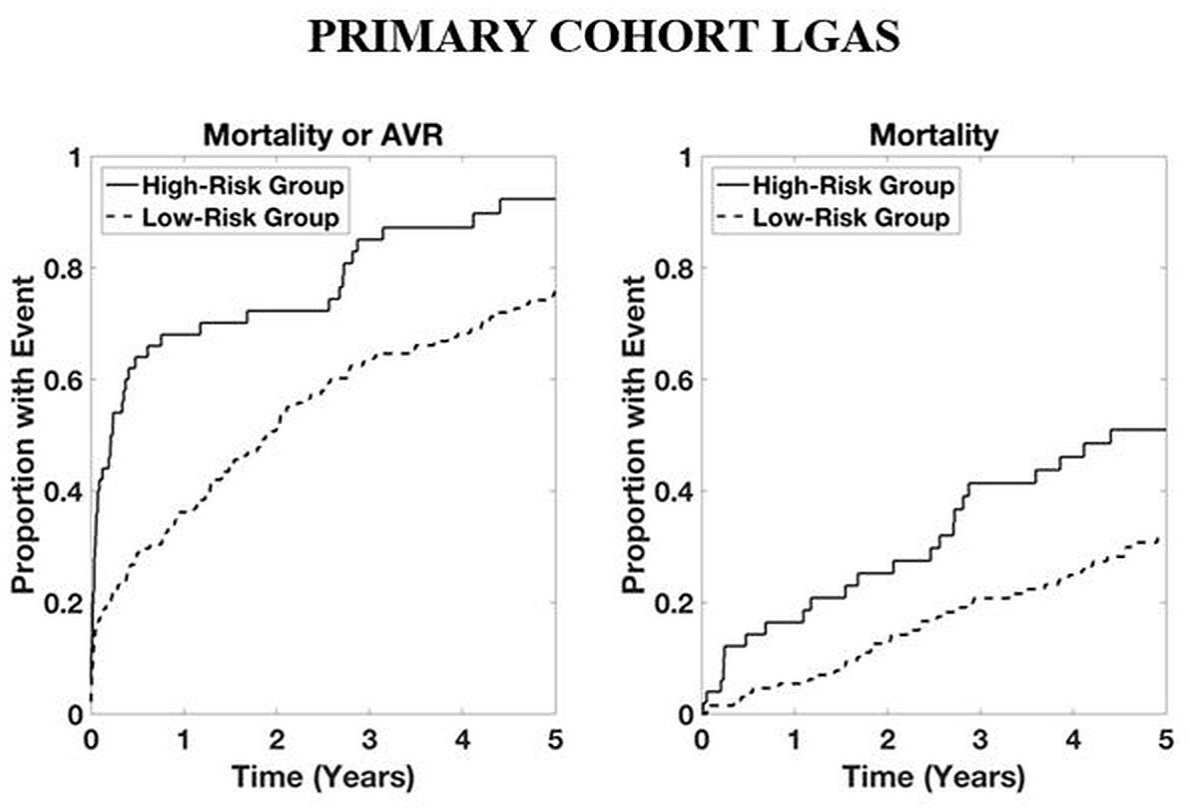
Time-to-event analysis in patients with low-gradient aortic stenosis (LGAS) within the primary cohort. High-risk group determined by upper quartile of ranked risk. Curves are averaged over 10 bootstrapped test sets. p<0.05 high-risk group versus others.

**Figure 4:**
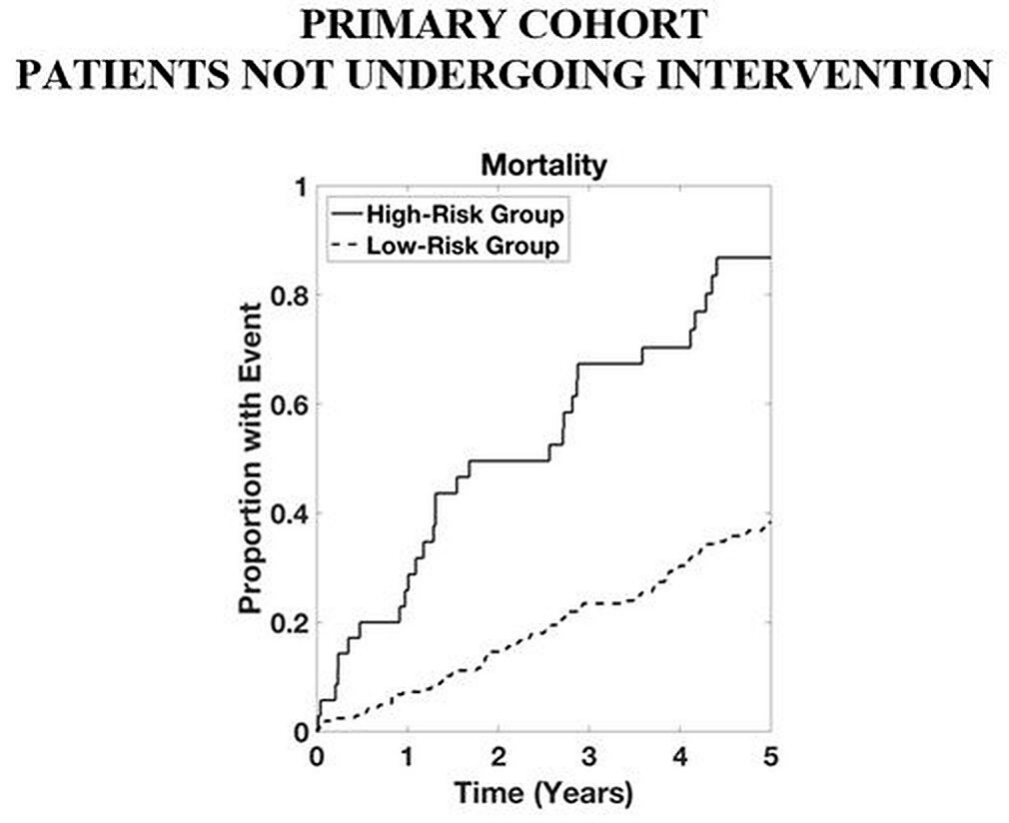
Time-to-event analysis in patients with aortic stenosis not undergoing an intervention within the primary cohort. High-risk group determined by upper quartile of ranked risk. Curves are averaged over 10 bootstrapped test sets. p<0.05 high-risk group versus others.

Time-to-event analyses for both the combined outcome and mortality alone are shown in figure 2. Time-to-event analyses for patients with LGAS and for patients not undergoing intervention are presented in figures 3 and 4, respectively. For both the combined outcome and mortality alone, both curves separate at 1 year, with differences between years 2 and 5 being statistically significant for predicting mortality.

**Figure 5:**
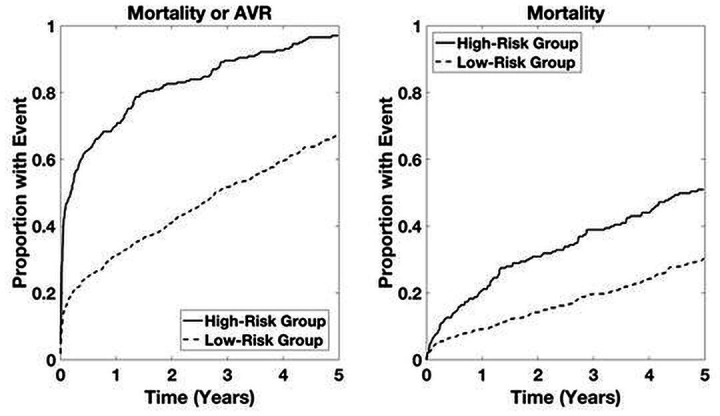
Time-to-event analysis based on ASteRisk score in patients with aortic stenosis within the validation cohort. High-risk group determined by upper quartile of ranked risk. p<0.05 high-risk group versus others. ASteRisk score, Aortic Stenosis Risk score.

Average risk of combined death/AVR in the risk quartiles is as follows: first quartile (lowest 25% of risk): 37.6%; second quartile: 55.2%; third quartile: 67.9%; fourth quartile (highest 25% of risk): 88.7%.

### Validation cohort

In the validation cohort, the ASteRisk score had superior discriminatory ability relative to that of the baseline model (table 2). The ASteRisk score also identified patients at high risk of death at years 1–5 in the entire validation cohort, LGAS cohort (n=316) and patients not undergoing intervention (n=196) (table 4). By contrast, the baseline model identified high-risk patients in the overall cohort and patients not undergoing an intervention at years 1–5, but not at any time point in the LGAS cohort (table 4).

**Table 4:** HRs for death in the validation cohort, LGAS subset and patients not undergoing intervention

HRs for death in the validation cohort (n=540)					
Model	1-Year HR (95% CI)	2-Year HR (95% CI)	3-Year HR (95% CI)	4-Year HR (95% CI)	5-Year HR (95% CI)
Baseline	2.1 (1.6 to 3.0)	1.9 (1.5 to 2.5)	1.9 (1.5 to 2.4)	1.9 (1.5 to 2.3)	1.8 (1.5 to 2.2)
ASteRisk score	2.6 (1.9 to 3.6)	2.6 (2.0 to 3.5)	2.6 (2.0 to 3.3)	2.4 (1.9 to 3.1)	2.4 (1.9 to 3.0)
**Patients with LGAS (n=316)**					
Baseline	1.0 (0.7 to 1.4)	1.0 (0.8 to 1.4)	1.1 (0.8 to 1.5)	1.1 (0.8 to 1.5)	1.2 (0.9 to 1.6)
ASteRisk score	2.3 (1.3 to 3.8)	2.6 (1.6 to 4.0)	2.5 (1.7 to 3.8)	2.5 (1.8 to 3.7)	2.5 (1.7 to 3.5)
**Patients without intervention (n=196)**					
Baseline	2.4 (1.5 to 3.9)	2.3 (1.6 to 3.5)	2.8 (2.0 to 3.9)	2.9 (2.2 to 4.0)	3.1 (2.4 to 4.2)
ASteRisk score	3.3 (2.0 to 5.3)	3.9 (2.6 to 5.7)	4.0 (2.8 to 5.6)	3.9 (2.8 to 5.4)	4.3 (3.1 to 5.9)

P value <0.05, where HR (95% CI) does not cross/include 1. HRs are calculated using the upper quartile of risk.

ASteRisk score, Aortic Stenosis Risk score; LGAS, low-gradient aortic stenosis.

**Figure 6:**
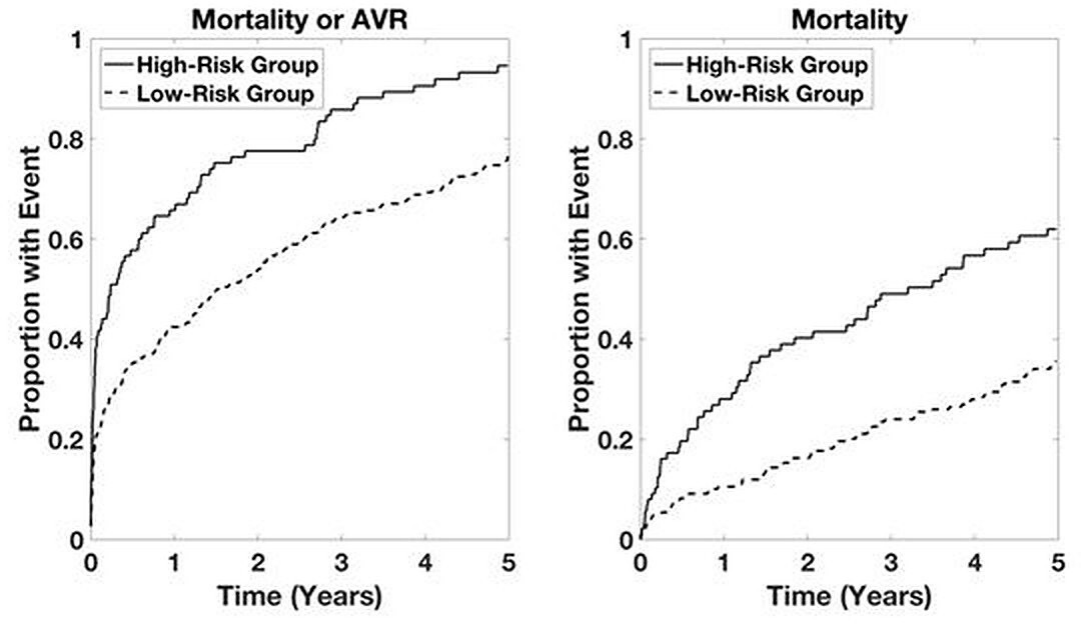
Time-to-event analysis in patients with low-gradient aortic stenosis (LGAS) within the validation cohort. High-risk group determined by upper quartile of ranked risk. p<0.05 high-risk group versus others.

**Figure 7:**
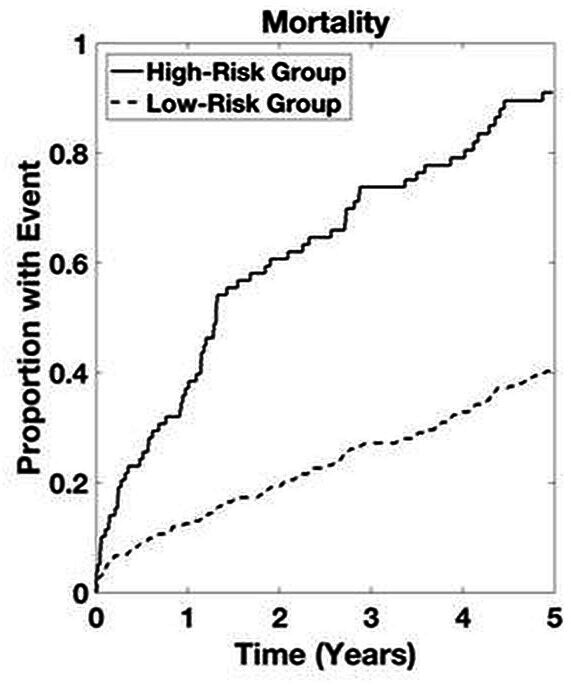
Time-to-event analysis in patients not undergoing an intervention within the validation cohort. High-risk group determined by upper quartile of ranked risk. p<0.05 high-risk group versus others.

Time-to-event analyses for all patients in the validation cohort are presented in figure 5 for the ASteRisk score. Time-to-event analyses for patients with LGAS and patients not undergoing intervention in the validation cohort are presented in figures 6 and 7, respectively. Again, both curves separate by 1 year, with differences between years 1 and 5 being statistically significant for predicting mortality.

## Discussion

**Figure 8:**
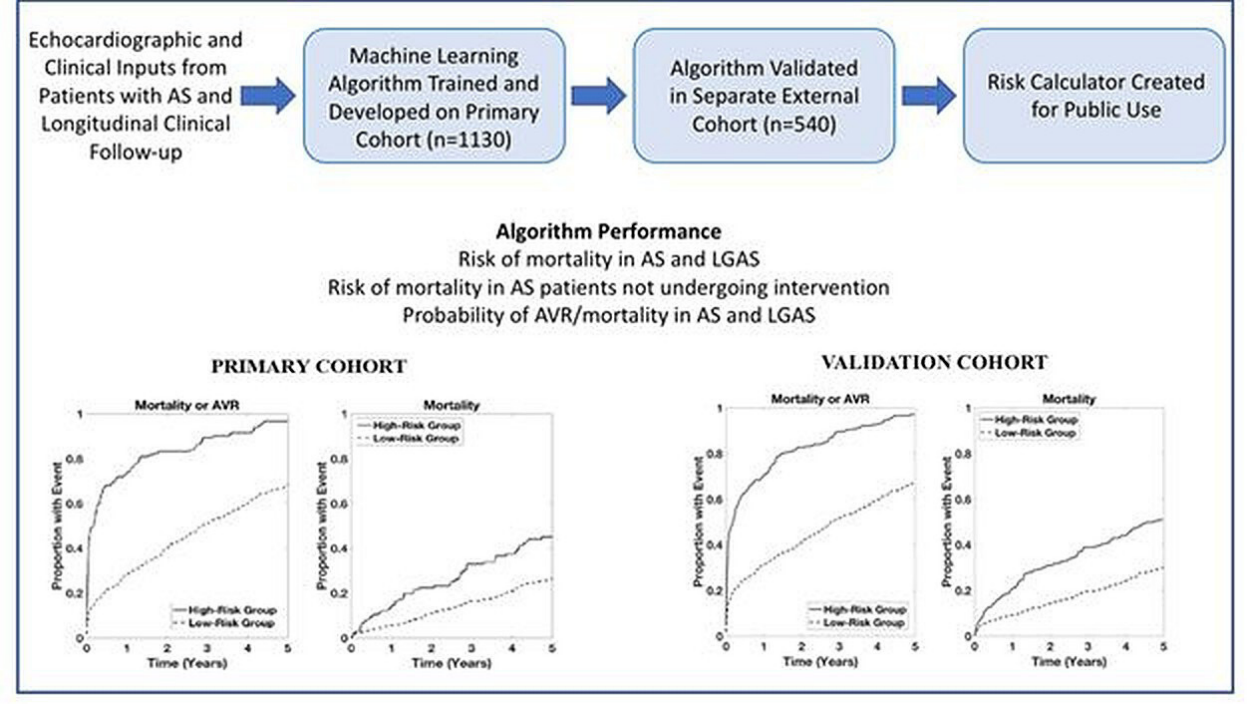
Training and validation of a machine learning algorithm that allowed prediction of mortality and AVR in patients with AS, including in LGAS. AS, aortic stenosis; AVR, aortic valve replacement; LGAS, low-gradient aortic stenosis.

Using two independent longitudinal databases from large tertiary hospitals, we have demonstrated that a machine learning algorithm using a small set of important features, readily available at the bedside, can be input to reliably calculate clinical risk in patients with aortic stenosis over long-term follow-up (figure 8). The algorithm outperforms a baseline risk model using conventional risk factors used to judge severe AS and also works in the traditionally challenging subgroup of LGAS. Moreover, the algorithm development process identified important features not traditionally considered in clinical risk assessment, but known from physiology to contribute to haemodynamic loading in AS.

A driving principle of this work was that a successful clinical risk model is not only determined by its performance but also by its ease of use.^8^ Although modern EHR systems can implement risk models that use an arbitrary number of features, such systems are not available in all clinical settings.^18^

Samad *et al*^5^ demonstrated good prediction capability (AUC 0.89) with a random forest model, but used echocardiography and clinical data to predict all-cause mortality in a general population. Our study focused on outcome within AS, and specifically also evaluated the extremely clinically challenging subgroup of LGAS. While the results of Samad *et al* are compelling, the models work at a population level, and teasing out specific risks in particular phenotypic subgroups is a different challenge altogether. This brings up the fundamental dilemma in machine learning applied to health, whereby there is a debate about the degree of ‘explainability’ a model needs.^8^ We take the view that if clinical practices are to be informed and influenced by machine learning models, and clinicians are to accept them, they first need to start with comprehensible models rather than broad ‘black-box’ approaches.

In addition to providing insight into how risks were determined, our model also permits some insights into the pathophysiology of AS. The set of features that appears in our predictive model includes both clinical (eg, heart failure) and echocardiographic (eg, mean gradient) variables that are known to have prognostic power. But interestingly, the model also identified transvalvular flow rate and transvalvular energy loss as important features. These findings are consistent with recent work highlighting the importance of flow rate in patients with AS^9 11 19^ which is not yet commonly used in current clinical practice. Transvalvular energy loss is considered the best measure of left ventricular afterload resultant from AS,^16 20 21^ and our algorithm’s selection of energy loss as a key determinant of outcome suggests that the concept of energy loss should be revisited in the assessment of AS. Furthermore, our findings suggest that echocardiographic approximations of energy loss are indeed clinically valuable and discriminatory for outcomes.^20 21^

The fact that our algorithm performs well on two large, independent datasets argues that it is indeed generalisable and therefore can be applied more widely to different patient cohorts. We therefore constructed a user interface that enables clinicians and researchers to easily use our algorithm. This online risk calculator, the MGH-MIT Aortic Stenosis Risk (ASteRisk) Calculator, is available at https://calc-as.herokuapp.com/. Our goal is to provide this tool for clinicians to use as an assistance in clinical decision-making in AS, but we recognise that it also provides an opportunity for prospective validation of our model across disparate geographic, demographic and healthcare settings globally. The calculator provides users with quantitative risk scoring based on our machine learning algorithm applied to the 9 bedside inputs.

Although the final ASteRisk score uses 9 inputs, it ultimately requires 11 features, all of which can be derived from the nine routine measurements that a clinician is required as input. Transvalvular flow rate and energy loss are derived from other inputs (figure 1).^11 16^ This was done because transvalvular flow rate and energy loss are not routinely measured during a routine echocardiographic study and we wanted inputs to be clinically accessible.

Furthermore, this algorithm does not require EHR infrastructure for clinicians to use, increasing the potential utility of our algorithm in a range of clinical settings where these technologies are unavailable or limited in their scope.^18^ We narrowed input number to 9 and there was no significant difference in model performance to a model with all 69 inputs.

Some limitations should be considered when interpreting our findings. We excluded patients with moderate or greater aortic or mitral regurgitation. Longitudinal data were retrospectively analysed. To achieve sufficient numbers to train a machine learning algorithm, it is necessary to use such retrospective data, but prospective validation would be important. The majority of patients in the primary cohort were Caucasian, making applicability to other demographics less certain.

Our data are only applicable to patients with moderate-to-severe AS. Patients with mild AS were excluded because they are unlikely to proceed to adverse clinical outcomes within 5 years of diagnosis.^22^

The outcome of AVR in the combined death/AVR is subject to clinical bias based on clinical decision-making to refer for and proceed with AVR—but nonetheless this is a commonly used outcome in AS studies. We also used all-cause mortality as a stand-alone outcome, which is less susceptible to bias.

We chose a BLR threshold of 85% (rather than 90%) to incorporate mean gradient as a feature. We did this because numerous studies have established the predictive power of mean gradient in AS^23–27^ and mean gradient is a central tenet of clinical AS risk assessment.^3 9^ We therefore believed that any model that left out mean gradient will be viewed with scepticism in the clinical community. Our cohort did have a large number of patients with LGAS and as such, this may have skewed findings to miss mean gradient as a selected feature using a 90% threshold. Nonetheless, we felt it important to include maintaining our model’s external validity and applicability across a range of cohorts, acknowledging our own data skew toward LGAS. We believed that the adjustment of BLR threshold by 5% was a small but necessary adjustment to permit inclusion of the important and universally recognised feature.

The recruitment periods for the primary and validation cohorts were different with respect to the ease of access and technical success rates with transcatheter aortic interventions which have improved considerably in the last decade. This may have had an effect on outcomes within the two cohorts.

Finally, our cohorts (and hence models) arise from two large tertiary referral centres in North America. The applicability to other settings must be considered in this context and further data in other populations and setting would be of value.

Using a machine learning algorithm, we were able to predict clinical outcome in two separate longitudinal cohorts for patients with AS, including in LGAS and patients not undergoing intervention. We provide an online risk calculator that permits the use of our algorithm for clinical and research purposes.

## Data Availability

Data are available on reasonable request. Due to institutional review board restrictions, we are unable to share the source data. The algorithm derived from these data is shared online for public use.
