# Supplementary material for: Predicting outcomes in patients with aortic stenosis using machine learning: the Aortic Stenosis Risk (ASteRisk) score
Source: Open Heart. 2022 May 31;9(1):e001990. doi: 10.1136/openhrt-2022-001990 (PMC9157386; doi:10.1136/openhrt-2022-001990)

Supplemental Appendix

Predicting Outcomes in Patients with Aortic Stenosis Using Machine Learning

Authors:

Mayooran Namasivayam, MBBS, PhD,[a] Paul D. Myers, MS,*[b] John V. Guttag, PhD,[b,e] Romain Capoulade, PhD,[c] Philippe Pibarot, DVM, PhD,[d] Michael H. Picard, MD,[a] Judy Hung, MD,**[a] Collin M. Stultz, MD, PhD,**[a,b,ff]*

** equal first authors*

*** equal senior authors*

Institutions:

a) *Division of Cardiology, Massachusetts General Hospital, Harvard Medical School, Boston, MA*
b) *Department of Electrical Engineering and Computer Science, Massachusetts Institute of Technology, Cambridge, MA*
c) *University of Nantes, CHU Nantes, CNRS, INSERM, l'institut du thorax, F-44000 Nantes, France*
d) *Quebec Heart and Lung Institute, Laval University, Quebec City, Canada*
e) *Computer Science and Artificial Intelligence Laboratory, Massachusetts Institute of Technology, Cambridge, MA.*
f) *Research Laboratory of Electronics, Massachusetts Institute of Technology, Cambridge MA.*

Table S1: Set of 69 features applied as input to the bootstrap lasso analysis along with the fraction of missing data for each.

Feature	Percent Missing
Body surface area	0.9%
Height	0.9%
Weight	0.9%
Gender	0.0%
Transvalvular flow rate	0.0%
Mean gradient	0.0%
Aortic valve area	0.0%
Bicuspid valve status	17.6%
Race	0.0%
Diabetes	0.0%
Hypertension	0.0%
Congestive heart failure	0.0%
Coronary artery disease	0.0%
Peripheral vascular disease	0.0%
Hyperlipidemia	0.0%
Atrial fibrillation	0.0%
Myocardial infarction	0.0%
Smoking status	14.6%
Beta blocker	0.0%
Angiotensin converting enzyme inhibitor	0.0%
Angiotensin receptor blocker	0.0%
Potassium sparing diuretics	0.0%
Dihydropyridine calcium channel blocker	0.0%
Nondihydropyridine calcium channel blocker	0.0%
Nitrate	0.0%

Statin	0.0%
Antiplatelet	0.0%
Oral Anticoagulant	0.0%
Chronic kidney disease	0.8%
Aortic sinus diameter	0.2%
Ascending aorta diameter	5.3%
Left atrial diameter	0.5%
Left ventricular end-diastolic dimension	0.1%
Left ventricular end-systolic dimension	0.1%
Ejection fraction without apical contribution	0.1%
Apical contribution to ejection fraction	7.3%
Estimated ejection fraction	0.1%
Ejection fraction measurement method	9.5%
Posterior wall thickness	0.3%
Interventricular septal thickness	0.2%
Peak trans-aortic valve gradient	0.0%
Aortic valve area indexed to body surface area	0.9%
Left ventricular outflow tract dimension	1.7%
Left ventricular outflow tract velocity	2.4%
Anterior-posterior left atrial dimension	41.2%
Superior-inferior left atrial dimension	41.1%
Medial-lateral left atrial dimension	42.3%
Estimated right ventricular systolic pressure	24.6%
Estimated right ventricular systolic pressure category	2.8%
Aortic valve calcification	23.6%
Aortic insufficiency	24.1%

Anterior mitral annulus calcification	29.8%
Mitral regurgitation	2.7%
Prolapse of anterior mitral leaflet	1.7%
Left atrial dilation	0.4%
Left ventricular cavity dilation	1.4%
Left ventricular systolic function	4.3%
Left ventricular hypertrophy	48.4%
Abnormal segmental wall motion	8.8%
Age	0.0%
Chronic kidney disease stage change event	26.2%
Diameter of aortic sinus of Valsalva level	0.0%
Area of aorta at sinus of Valsalva level	0.0%
Energy loss coefficient	0.0%
Peak velocity	0.0%
Energy loss method 1 using peak velocity	0.2%
Dimensionless velocity index	0.0%
Energy loss method 2 using flow rate	0.2%
Valve resistance	0.0%

Table S2: Hazard ratios for mortality in the different subsets of patients not undergoing intervention (n=776)*. Hazard ratios are calculated using the upper quartile of risk. $p<0.05$ where HR 95% CI does not cross/include 1. Abbreviations: AVA = aortic valve area; MG: mean gradient.

Outcome	1-Year HR (95% CI)	2-Year HR (95% CI)	3-Year HR (95% CI)	4-Year HR (95% CI)	5-Year HR (95% CI)
AVA ≤ 1.0 cm^2 and MG ≥40 mmHg (n=32)	1.5 (0.2, 12.7)	1.8 (0.2, 14.3)	2.0 (0.3, 15.9)	2.3 (0.3, 16.1)	2.3 (0.4, 16.2)
AVA ≤ 1.0 cm^2 (n=334)	3.0 (1.4, 6.5)	3.0 (1.7, 5.4)	2.7 (1.7, 4.5)	2.5 (1.5, 3.9)	2.4 (1.6, 3.8)
AVA >1.0 cm^2 and MG <40 mmHg (n=440)	6.1 (0.8, 47.2)	4.9 (0.9, 28.8)	4.8 (1.0, 25.7)	4.5 (0.9, 24.0)	4.4 (0.9, 23.3)

*while 776 patients in the primary cohort did not undergo intervention, the subgroups listed in this table represent n=774 because two patients had AVA >1 and MG ≥ 40 mmHg and are not included as a subgroup here.

Table S3: Performance of model for the outcome of AVR in the primary and validation cohorts, including in patients with LGAS. Hazard ratios are calculated using the upper quartile of risk. $p<0.05$ where HR 95% CI does not cross/include 1.

Abbreviations: AVR = aortic valve replacement; LGAS: low-gradient aortic stenosis.

Outcome	1-Year HR (95% CI)	2-Year HR (95% CI)	3-Year HR (95% CI)	4-Year HR (95% CI)	5-Year HR (95% CI)
**Primary Cohort (n=1130)**					
AVR	8.6 (3.5, 20.8)	6.0 (3.1, 11.7)	3.8 (2.2, 6.5)	2.9 (1.8, 4.6)	2.4 (1.6, 3.7)
**LGAS - Primary Cohort (n=383)**					
AVR	3.7 (0.7, 22.8)	3.5 (0.7, 19.5)	1.8 (0.7, 4.6)	1.3 (0.6, 3.1)	1.4 (0.6, 2.9)
**Validation Cohort (n=540)**					
AVR	4.1 (3.1, 5.5)	3.9 (3.0, 5.0)	3.6 (2.9, 4.6)	3.4 (2.7, 4.3)	3.5 (2.8, 4.4)
**LGAS – Validation Cohort (n=316)**					
AVR	2.3 (1.4, 3.6)	1.9 (1.3, 3.0)	2.0 (1.3, 2.9)	1.9 (1.2, 2.7)	1.9 (1.3, 2.8)

Table S4: Individual area under the curve for 9 features of final model.

Flow rate only: 0.54+/-0.03
Mean gradient only: 0.59+/-0.02
AVA only: 0.64+/-0.03
Energy loss only: 0.66+/-0.03
Hyperlipidaemia only: 0.52+/-0.02
Posterior wall thickness only: 0.62+/-0.03
CKD only: 0.55+/-0.03
CHF only: 0.60+/-0.03
MI OR PVD OR Wall motion abnormality only: 0.55+/-0.03

Abbreviations: AVA: aortic valve area; CHF: congestive heart failure; CKD: chronic kidney disease; MI: myocardial infarct; PVD: peripheral vascular disease.

Table S5: Baseline characteristics by aortic valve replacement status in primary cohort, reported as median (IQR).

	**No AVR**	**AVR**	**Sig.***
**Age**	80 (72-86)	75 (66-81)	<0.001
**Mean gradient (mmHg)**	23 (19-30)	33 (25-46)	<0.001
**Aortic valve area (cm^2)**	1.1 (0.9-1.3)	0.9 (0.8-1.1)	<0.001

* Using Mann-Whitney U test

Figure S1: Time to event analysis based on machine learning based prediction model of risk in aortic stenosis in all patients within the primary cohort. High-risk group determined by upper quartile of ranked risk. Abbreviations: AVR: aortic valve replacement. Curves are averaged over 10 bootstrapped test sets. p<0.05 high risk group vs. others.

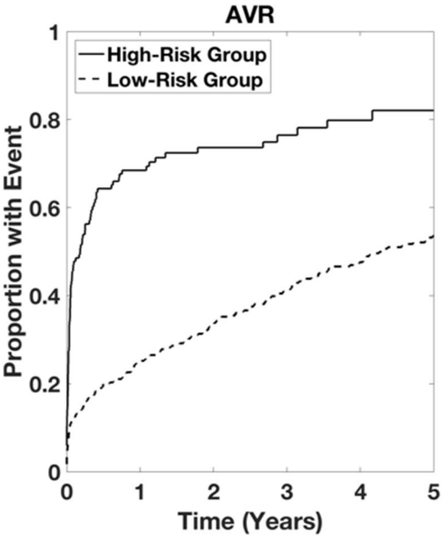

Figure S2: Time to event analysis based on machine learning based prediction model of risk in aortic stenosis in patients with low-gradient aortic stenosis (LGAS) within the primary cohort. High-risk group determined by upper quartile of ranked risk. Abbreviations: AVR: aortic valve replacement. Curves are averaged over 10 bootstrapped test sets. p<0.05 high risk group vs. others.

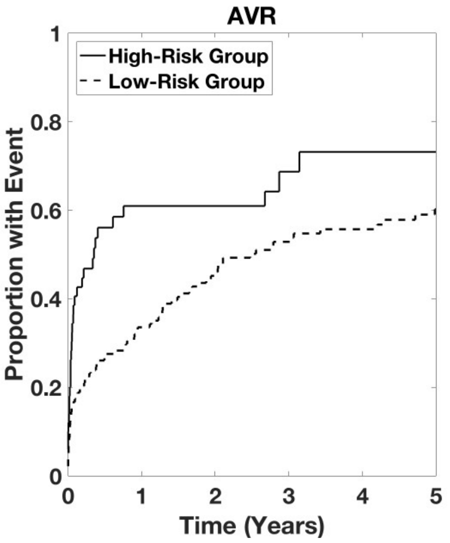

Figure S3: Time to event analysis based on machine learning based prediction model of risk in aortic stenosis in all patients within the validation cohort. High-risk group determined by upper quartile of ranked risk. Abbreviations: AVR: aortic valve replacement. p<0.05 high risk group vs. others.

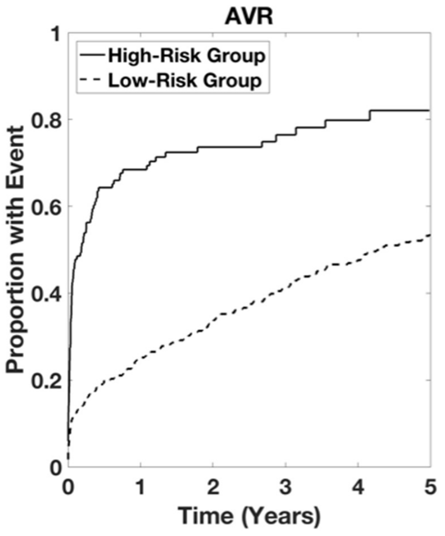

Figure S4: Time to event analysis based on machine learning based prediction model of risk in aortic stenosis in patients with low-gradient aortic stenosis (LGAS) within the validation cohort. High-risk group determined by upper quartile of ranked risk. Abbreviations: AVR: aortic valve replacement. $p<0.05$ high risk group vs. others.

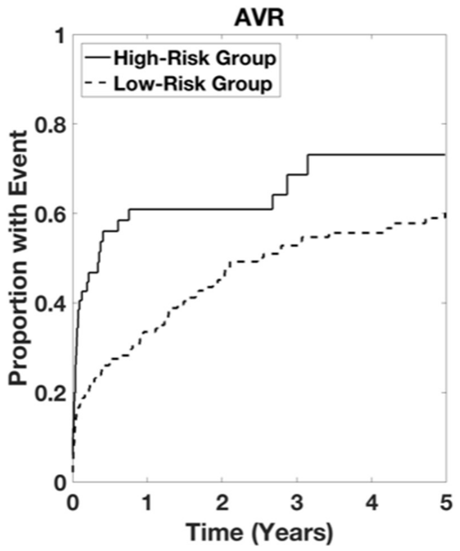

Figure S5: Distributions of Massachusetts General Hospital (MGH) and Laval (Quebec) cohorts (n) based on aortic valve area (AVA) (cm^2).

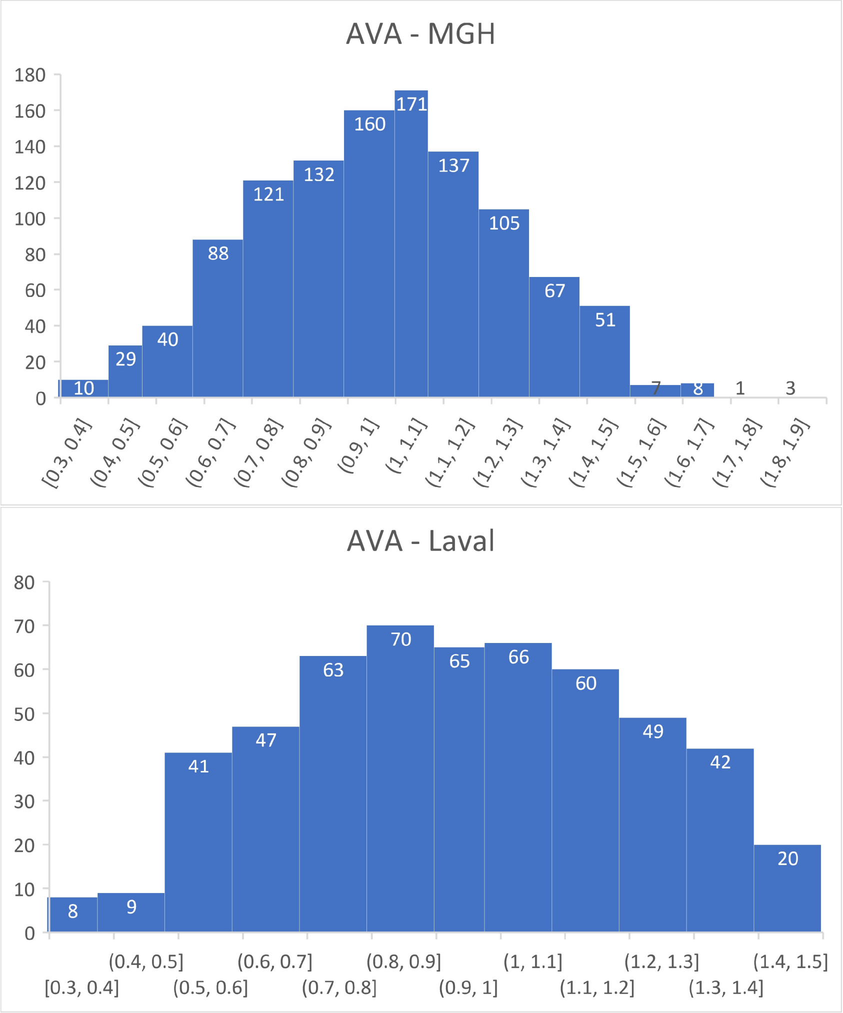

Figure S6: Distributions of Massachusetts General Hospital (MGH) and Laval (Quebec) cohorts (n) based on mean gradient (mmHg).

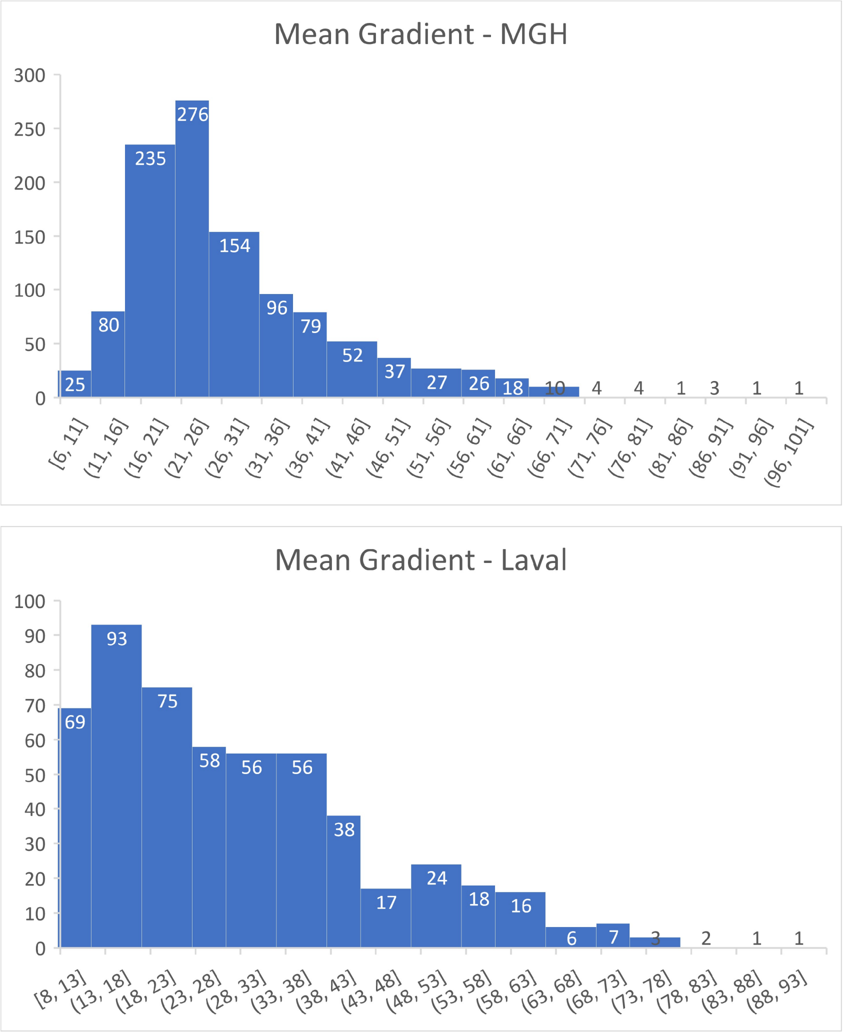

Figure S7: Waterfall plot outlining individual features contributing to risk in a given patient. Red indicates increased risk, blue indicates reduced risk, relative magnitude of risk indicated by x-axis. Features: 3) congestive heart failure, 2) aortic valve area </= 1.0 cmsq, 7) posterior wall thickness, 1) mean gradient >/= 40 mmHg, 6) chronic kidney disease, 5) hyperlipidaemia, 4) myocardial infarct, peripheral vascular disease or regional wall motion abnormality, 8) energy loss, 0) transvalvular flow rate.

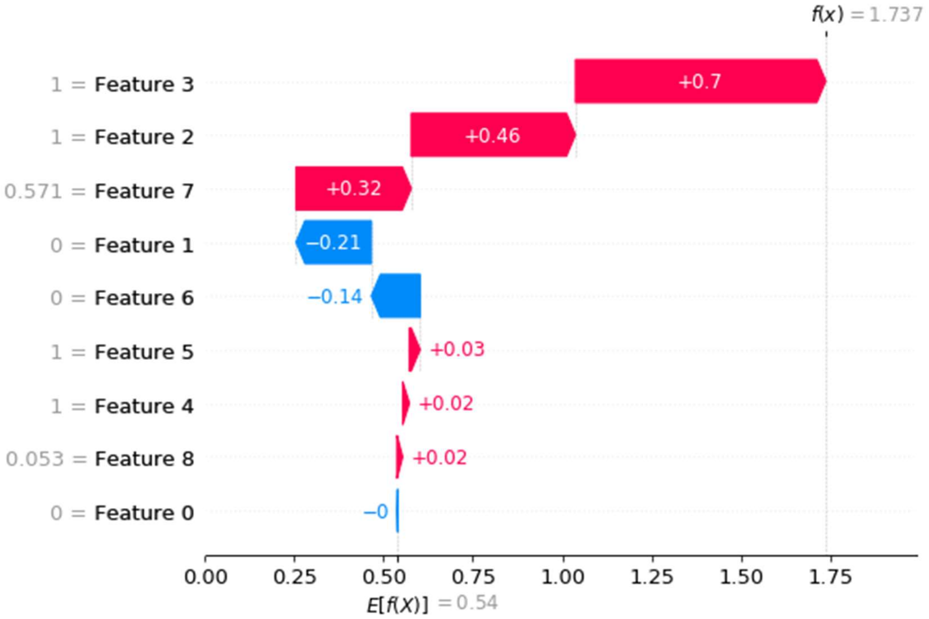

Supplement: Supplementary data [file openhrt-2022-001990supp001.pdf]
